# Development of morbidity and mortality of SARS-CoV-2 in nursing homes for the elderly in Frankfurt am Main, Germany, 2020–2022: What protective measures are still required?

**DOI:** 10.3205/dgkh000431

**Published:** 2023-02-14

**Authors:** Ursel Heudorf, Eugen Domann, Markus Förner, Sabine Kunz, Leo Latasch, Bernd Trost, Katrin Steul

**Affiliations:** 1 Justus Liebig University Giessen, Giessen, Germany; 2 Hufeland-Haus, Frankfurt am Main, Germany; 3 August-Stunz-Zentrum, Frankfurt am Main, Germany; 4 Altenzentrum der Jüdischen Gemeinde, Frankfurt am Main, Germany; 5 Franziska-Schervier Seniorenzentrum, Frankfurt am Main, Germany; 6 Johannes Gutenberg University Mainz, Mainz, Germany

**Keywords:** SARS-CoV-2, COVID-19, nursing-home residents, hospitalization, case fatality rate, pandemic

## Abstract

**Introduction:**

Nursing-home residents are among the highest risk group in the SARS-CoV-2 pandemic. At the onset of the SARS-CoV-2 pandemic, the majority of all deaths from or with SARS-CoV-2 occurred in long-term care facilities (LTCFs), so that maximum protective measures were mandated for these facilities. This study analyzed the impact of the new virus variants and the vaccination campaign on disease severity and mortality among nursing home residents and staff through 2022 as a basis for determining which protective measures remain necessary and appropriate.

**Methods:**

In five homes in Frankfurt am Main, Germany, with a total capacity for 705 residents, all cases occurring in the facility among residents and staff were recorded and documented (date of birth and diagnosis, hospitalization and death, vaccination status) and were descriptively analyzed with SPSS.

**Results:**

By 31^st^ August 2022, 496 residents tested positive for SARS-CoV-2, 93 in 2020, 136 in 2021, and 267 in 2022; 14 residents presented with a second SARS-CoV-2 infection in 2022, having previously experienced an infection in 2020 or 2021. The percentage of hospitalizations decreased from 24.7% (2020) and 17.6% (2021) to 7.5% (2022), and the percentage of deaths decreased from 20.4% and 19.1% to 1.5%. In 2021, 61.8% of those infected were vaccinated (at least 2x); in 2022, 86.2% of residents had been vaccinated twice, 84% of whom had already had a booster vaccination. Hospitalization and death rates were significantly higher among the unvaccinated than the vaccinated throughout all years (unvaccinated 21.5% and 18.0%; vaccinated 9.8% and 5.5%; KW test p=0.000). However, this difference was no longer significant under the prevalence of the Omicron variant in 2022 (unvaccinated 8.3% and 0%; p=0.561; vaccinated 7.4% and 1.7%; p=0.604). From 2020 to 2022, 400 employees were documented as infected, with 25 having second infections in 2022. Only one employee showed a second infection in 2021 following the first in 2020. Three employees were hospitalized; no deaths occurred.

**Discussion and conclusion:**

Severe COVID-19 courses occurred with the Wuhan Wild type in 2020, with a high death rate among nursing-home residents. In contrast, during the waves in 2022 with the relatively mildly pathogenic Omicron variant, many infections but few severe courses and deaths were observed among the now mostly vaccinated and boostered nursing-home residents. Given the high immunity of the population and the low pathogenicity of the circulating virus – even in nursing-home residents – protective measures in nursing homes that restrict people’s right to self-determination and quality of life no longer seem justified. Instead, the general hygiene rules and the recommendations of the KRINKO (German Commission for Hospital Hygiene and Infection Prevention) on infection prevention should be followed, and the recommendations of the STIKO (German Standing Commission on Vaccination) on vaccination not only against SARS-CoV-2 but also against influenza and pneumococci should be observed.

## Zusammenfassung

**Einleitung:**

Altenpflegeheimbewohner zählen zu der höchsten Risikogruppe in der SARS-CoV-2-Pandemie. Zu Beginn der SARS-CoV-2-Pandemie traten die Mehrzahl aller Todesfälle an oder mit SARS-CoV-2 in Altenpflegeheimen (APHs) auf, weshalb für die Einrichtungen maximale Schutzmaßnahmen verfügt wurden. Die Auswirkungen der neuen Virus-Varianten und der Impfkampagne auf die Krankheitsschwere und Sterberate bei Altenpflegeheimbewohnern bis 2022 wird untersucht – als Grundlage für die Frage, welche Schutzmaßnahmen weiterhin erforderlich und angemessen sind.

**Methode:**

In fünf Heimen in Frankfurt am Main mit insgesamt 705 Bewohnerplätzen wurden alle in der Einrichtung auftretenden Fälle sowohl bei Bewohnern als auch bei Mitarbeitern erfasst und dokumentiert (Geburts- und Diagnosedatum, Hospitalisierung und Tod, Impfstatus) und mit SPSS deskriptiv ausgewertet.

**Ergebnisse:**

Bis August 2022 wurden 496 Bewohner positiv auf SARS-CoV-2 getestet, 93 im Jahr 2020, 136 im Jahr 2021 und 267 im Jahr 2022. 14 Bewohner wiesen im Jahr 2022 eine zweite SARS-CoV-2-Infektion auf, nachdem sie bereits 2020 oder 2021 eine Infektion durchgemacht hatten. Der Anteil der Hospitalisierungen nahm von 24,7% (2020) über 17,6% (2021) auf 7,5% (2022) ab, der Anteil der Todesfälle von 20,4% über 19,1% auf 1,5%. Im Jahr 2021 waren 61,8% der Infizierten geimpft (mindestens 2x), im Jahr 2022 waren 86,2% der Bewohner geimpft, 84% davon bereits „geboostert“. Die Hospitalisierungs- und Todesrate war bei den Ungeimpften in der Gesamtbetrachtung signifikant höher als bei den Geimpften (Ungeimpfte 21,5% und 18,0%; Geimpfte 9,8% und 5,5%; KW Test p=0,000), allerdings war dieser Unterschied unter dem Vorherrschen der Omikron-Variante im Jahr 2022 nicht mehr signifikant (Ungeimpfte 8,3% und 0%; p=0,561; Geimpfte 7,4% und 1,7%; p=0,604). Von 2020 bis 2022 wurden 400 Mitarbeiter als infiziert dokumentiert, 25 Mitarbeiter wiesen eine Zweitinfektion im Jahr 2022 auf. Nur bei einem Mitarbeiter war die zweite Infektion bereits 2021 aufgetreten, nach der ersten im Jahr 2020. Drei Mitarbeiter wurden hospitalisiert, Todesfälle traten nicht auf.

**Diskussion und Schlussfolgerung:**

2020 kam es unter dem Wuhan-Wild-Typ zu schweren COVID-19-Verläufen mit einer hohen Todesrate unter den Altenpflegeheimbewohnern. Demgegenüber waren während der Wellen in 2022 mit der wenig pathogenen Omicron-Variante bei den inzwischen zumeist geimpften und geboosterten Altenpflegeheimbewohnern zwar viele Infektionen aber nur wenige schwere Verläufe und Todesfälle zu beobachten. Angesichts der hohen Immunität der Bevölkerung und der geringen Pathogenität des zirkulierenden Virus – auch bei Altenpflegeheimbewohnern – erscheinen. Schutzmaßnahmen in Altenpflegeheimen, die das Selbstbestimmungsrecht und die Lebensqualität der Menschen einschränken, nicht mehr gerechtfertigt. Stattdessen sollten die allgemeinen Hygieneregeln und die Empfehlungen der KRINKO (Kommission für Krankenhaus-hygiene und Infektionsprävention) zur Infektionsprävention umgesetzt sowie die Empfehlungen der STIKO (Ständige Impfkommission) zur Impfung nicht nur gegen SARS-CoV-2 sondern auch gegen Influenza und Pneumokokken beachtet werden.

## Introduction

In late 2019, first cases of a new pandemic virus, later named SARS-CoV-2, occurred in China. In March 2020, World Health Organization declared it a pandemic. Since then 623,893,894 infections have been reported and 6,553,936 deaths from or with COVID-19 have been recorded worldwide through Oct. 21, 2022 [1]. In Germany, the number of those reported with a positive SARS-CoV-2 test was 35,098,062 and deaths were 152,278 by 10/21/2022 [2]. In Germany, the case-fatality rate was 5.7% in June 2020, but since May 2022, it has been about 0.5%, and still decreasing (Oct. 21, 2022: 0.43%) [3]. However, all these figures are strongly influenced by test availability, test regime and reporting (completeness of data sets?). In addition, the case-fatality ratio depends on the pathogenicity of the prevailing virus variant and the progress of vaccination campaigns. 

It was already evident in March/April 2020 that the highest risk of morbidity and mortality affected the elderly, especially nursing-home residents. Although nursing-home residents make up only about 1% of the population in many countries, 30–70% of the deceased were nursing-home residents [4], [5]. Therefore, rigorous measures were imposed on nursing homes to protect this at-risk group, with bans on visits, strict hygiene, isolation, and quarantine measures. As a result, considerable “collateral damage” was observed in nursing home residents, including loneliness, depression, loss of quality of life and will to live, although in some cases residents were reported to show good resilience [6], [7], [8], [9], [10], [11], [12], [13], [14], [15], [16]. Visitation bans were gradually mitigated into visit restrictions, accompanied by extensive testing obligations for visitors and staff. Once vaccine was available, vaccination was recommended as a priority to the very elderly, nursing-home residents, and their carers (caregivers and family members) [17]. This offer was well-accepted: already by the end of 2021, 93% of residents and 86% of staff in nursing homes in Germany had been fully vaccinated (2x) and 70% of residents and 51% of staff had received the booster vaccination recommended only a few weeks earlier. By summer 2022, 94% of residents and staff had been vaccinated 2x and 85% of residents as well as 72% of staff had been vaccinated 3x [18], [19].

By October 2022, several “Variants of Concern” (VOC) of SARS-CoV-2 had emerged worldwide, triggering further pandemic waves. In Germany, 6 pandemic waves have been described to date: The first and second pandemic waves (calendar weeks 10–20/2020 and calendar weeks 40/2020–8/2021) were caused by the wild virus, the third wave (calendar weeks 9–23/2021) by the alpha variant, the fourth wave (calendar weeks 31–51/2021) by the delta variant, the fifth wave (CW 52/2021–CW 21/2022) by the omicron variant with sublines BA1 and BA2, and since summer 2022 (start of wave 6), subline BA5 of the omicron variant now predominates [20], [21]. In particular, the highly contagious Omicron variant BA1, BA2, and BS5 led to a maximum increase in the number of new infections in 2022 in all populations, both vaccinated and unvaccinated. 

Comparable trends were observed in other countries, which is why some countries declared the end of the pandemic, and ended pandemic measures in response to the low pathogenicity of the variants circulating as well as the high immunological response of the population. In Germany, however, the Infection Protection Act was extended in September 2022 and special protective measures for medical and nursing facilities and the vulnerable groups being cared for in these facilities have been extended until March 2023, in addition to the obligation to wear FFP2 masks in long-distance trains. In medical and nursing facilities, FFP2 masks are now mandatory and employees are required to test themselves 3 times a week [22], even though there is insufficient epidemiological evidence for this measure [23]. 

The Robert Koch Institute (RKI) currently (October 2022) continues to recommend 10 days of isolation for asymptomatic nursing-home residents testing positive for SARS-CoV-2. According to the RKI, SARS-CoV-2 infected, symptomatic nursing-home residents must be symptom-free for at least 2 days or at least have sustained clinical improvement, usually have been isolated and finally tested negative for 14 days before they can be released from isolation again [24]. Only for special individual situations (e.g., palliative care) does the RKI refer to possible exceptions, e.g., the recommendations of the German Society for Palliative Medicine [25] and the S1 guideline of the German Consortium of scientific societies [26]. 

There was general agreement on the need for strong protective measures for the vulnerable group of nursing-home residents at the beginning of the pandemic [27], [28], [29], [30], [31], [32], [33], [34]. Meanwhile, the massive unintended side-effects suffered by nursing-home residents have become well known. Today, nursing-home residents and their caregivers have a very high vaccination rate; and the currently dominant virus variant is significantly less pathogenic than the virus wild type. Thus, the question arises as to whether or which measures remain necessary and appropriate to protect nursing-home residents. In this context, SARS-CoV-2 infections and case fatality rates in nursing homes for the elderly from the beginning of the pandemic until summer 2022 is analyzed and discussed below. 

## Materials and methods

In five homes in Frankfurt am Main with a total capacity of 705 residents, all SARS-CoV-2 cases (residents and staff) documented in the facility were recorded (date of birth and diagnosis, hospitalization and death, if applicable, vaccination status) and analyzed descriptively with SPSS. Hospitalizations were documented only if they occurred because of COVID-19 and not for other reasons (e.g., injuries from falls or similar). Vaccinations were included in the calculations only if they had been received at least 14 days prior to infection. Fatalities with dates of death up to 30 days after the onset of illness or positive PCR test were documented as COVID-19-associated. 

Additionally, days of care and deaths for all residents from 2020 to 2022 (2022 to Aug. 31, 2022) were recorded. Calculations were performed with the SPSS program, including significance calculations (Kruskal-Wallis test and Mann-Whitney test).

Data on the prevalence of the different variants in Germany were retrieved from the RKI homepage [20] as well as the 7-day reporting data/100,000 population in Frankfurt am Main [35]. 

## Results

Table 1 (Tab. 1) shows total days of care, deaths (as total but also per 1,000 nursing days ND), SARS-CoV-2 infections and SARS-CoV-2-associated deaths per year. Between 2020 and 2021, care days decreased and deaths increased. From 2020 to Aug. 31, 2022, SARS-CoV-2 infections increased significantly, but SARS-CoV-2-associated deaths decreased significantly. From 2020 to 2022, based on 1,000 days of care, SARS-CoV-2 infections increased from 0.340/1,000 ND to 1.412/1,000 ND, and SARS-CoV-2 associated deaths decreased from 0.069/1,000 ND to 0.021/1,000 ND. Mortality from or with COVID-19 decreased from approximately 20% in 2020 and 2021 to 1.5% in 2022. Whereas in 2020 and 2021, about one in 10 deaths was from or with SARS-CoV-2, in 2022, the proportion of SARS-CoV-2-associated deaths was 2.7%, despite the very high rate of infection in 2022. 

Table 2 (Tab. 2) lists the cases of SARS-CoV-2 infection among residents and staff in the different years, including information on typical SARS-CoV-2 symptoms and hospitalization or death in connection with the SARS-CoV-2 infection, as well as information on the vaccination status at the time of infection (i.e., last vaccination at least 14 days before SARS-CoV-2 detection or symptom onset). Among residents, infections increased from 93 in 2020 to 267 in 2022. The mean age of the residents infected was 82.9 years, with no significant differences between the years. In all years, only slightly more than one in three residents with positive SARS-CoV-2 detection had presented indicative symptoms. The hospitalization rate decreased from 24.7% to 17.6% to 7.5% from 2020 to 2021 and to 2022, and the case-fatality rate decreased from approximately 20% in 2020 and 2021 to 1.5% in 2022. While none of the infected residents had been vaccinated in 2020, a total of 31.6% of those infected had not been vaccinated in 2021, 61.8% had been vaccinated twice, and 6.6% had been vaccinated three times. In 2022, approximately 13% each were unvaccinated or vaccinated only 2x, while 73% had already received vaccination three times. Hospitalization and death rates were significantly higher among the unvaccinated than the vaccinated overall (unvaccinated 21.5% and 18.0%; vaccinated 9.8% and 5.5%; KW test p=0.000), but this difference was no longer significant when the Omicron variant was prevalent in 2022 (unvaccinated 8.3% and 0%; p=0.561; vaccinated 7.4% and 1.7%; p=0.604). Moreover, hospitalization and death rates in residents vaccinated three times did not differ from the hospitalization and death rates of unvaccinated residents (MW-test p=0.643 and p=0.484).

Among employees, infection rates also increased sharply from 2020 to 2022, from 66 in 2020 to 255 in the first 8 months of 2022. Whereas approximately 45% of employees had complained of symptoms characteristic of SARS-CoV-2 in 2020 and 2021, the percentage was 56.5% in 2022. Hospitalization rates were very low in all years, and no employee died from or with SARS-CoV-2. In 2021, 54% of those infected with SARS-CoV-2 were unvaccinated and 45.6% were vaccinated; in 2022, 9.4% of them were unvaccinated, 34.1% were doubly vaccinated, and 56.5% were triply vaccinated. Neither overall from 2020 to 2022 nor in 2022 alone were significant differences detected in hospitalization rates between vaccinated and nonvaccinated employees.

**Figure 1:**
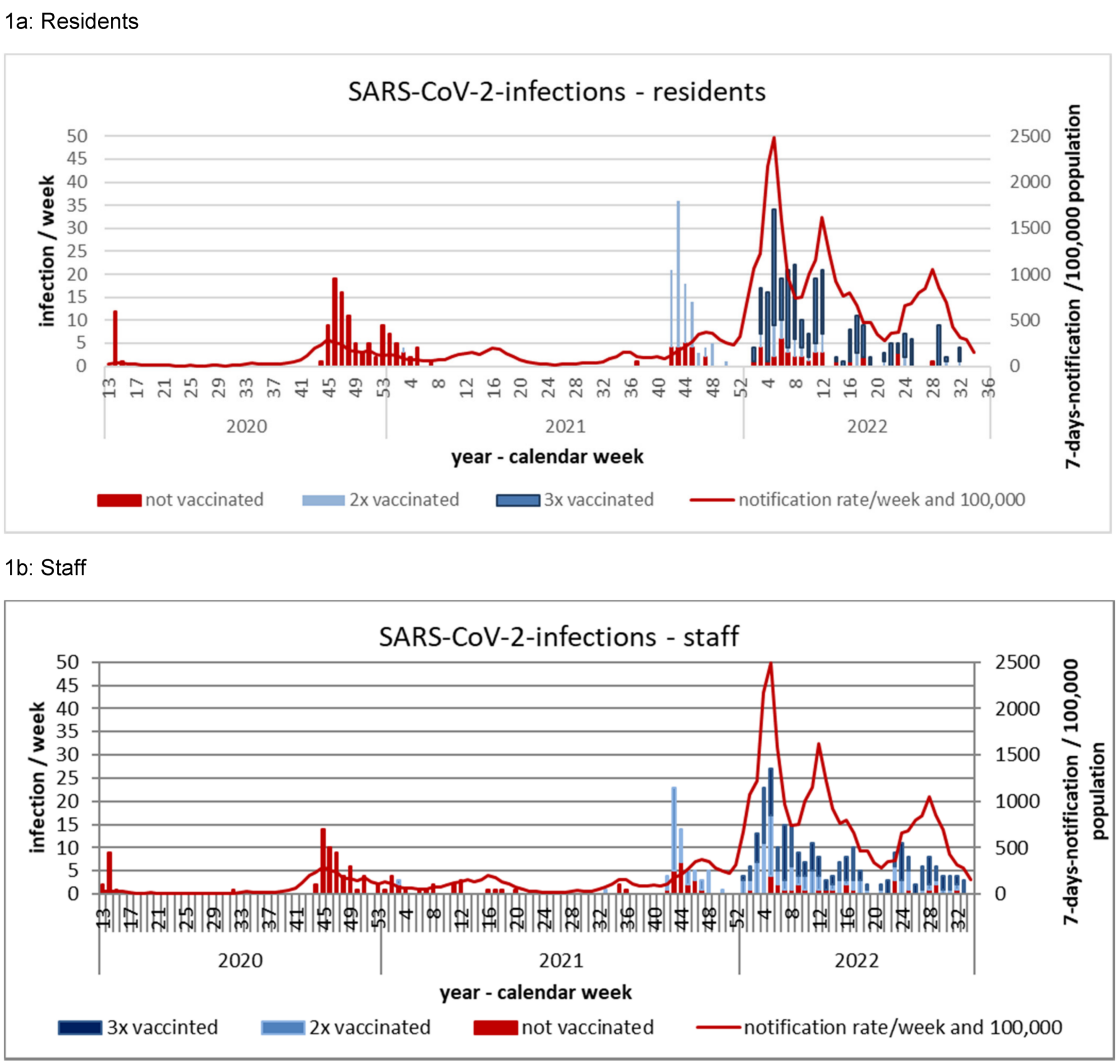
Weekly SARS-CoV-2 infections in residents (1a) and staff (1b) of 5 long-term care facilities in the city of Frankfurt am Main, Germany, March 2020–August 2022 compared to the 7-days notification rate/100,000 in the total population of the city

Figure 1 (Fig. 1) shows new infections per calendar week in relation to vaccination status for residents (Figure 1a (Fig. 1)) and staff (Figure 1b (Fig. 1)) – compared with the 7-day reporting rate of SARS-CoV-2 new infections in the population of Frankfurt am Main. 

## Discussion

By 2022, the corona pandemic in Germany had evolved in five waves. The first two waves in 2020 were caused by the wild virus, and the third and fourth waves in 2021 were caused by two variants of concern (Alpha and Delta). In 2022, additional waves were due to the omicron variant, with its subtypes BA1, BA2, and BA5 [20]. While the 7-day reporting rate (n/100,000) in the first two waves reached a maximum value in the second wave of 257/100,00 (wild virus), 198/100,000 in the third wave (alpha variant), and 368/100,000 in the fourth wave (delta variant), infections during the predominance of the omicron variant increased rapidly to maximum values up to 2489/100,000 in Germany. While testing capacities were limited in the first and second waves, there were adequate resources from 2021 onward. Because the testing regimen was largely identical in 2021 and 2022, there is no significant test bias to be considered in the extreme increase in infections in 2022. This increase in infections under omicron occurred despite the fact that by the end of December 2021, approximately 70% of the population was already vaccinated (twice) and approximately 40% had received the booster vaccination.

Infections and outbreaks have also occurred in nursing homes in Germany during the various pandemic waves [36], [37], [38], [39], [40]. While most publications found that direct transmissions between persons (staff, visitors, residents) were causative, in some outbreaks, ventilation (faults in the technical ventilation system, use of recirculated air or insufficient window ventilation) was involved [41], [42], [43], [44], [45], [46]. In the homes presented here, an outbreak was quickly terminated in the first wave. In the second wave, most facilities were affected. The third wave hardly impacted nursing-home residents who had already been vaccinated; however, some infections occurred among staff, but this did not result in further outbreaks in the facilities. Further infections among staff detected through regular mandatory staff testing also did not lead to any transmission to residents in the spring and summer of 2021. In the fourth wave, probably due to declining vaccine protection [47], [48], numerous infections and outbreaks occurred again in elderly-care facilities. Prompt booster vaccinations of nursing-home residents and staff, recommended by the STIKO [49], quickly initially stopped further spread, but did not prevent an extreme increase in infections with the new omicron variant in 2022. Within 8 months, far more residents and staff became infected than in the entire 2 preceding years. The testing regime in nursing homes had not changed significantly from 2021 to 2022: Staff members were and are obliged to test themselves several times a week before starting duty, and residents were and are tested when they have indicative symptoms, after exposure, or in connection with outbreak investigations. 

The omicron variant is significantly more contagious than the previous variants, but much less pathogenic [20]. The lower pathogenicity was also evident among nursing-home residents in Frankfurt. Although the hospitalization rate had decreased somewhat in 2021 compared with the previous year, the case fatality rate was approximately 20% in both years. In contrast, in 2022, the hospitalization rate decreased to one-third and the case fatality rate decreased to less than one-tenth of the previous values, to 1.5%. Comparable case fatality rates were also reported from Munich: By the beginning of 2022, the case fatality rate among nursing-home residents in Munich was 18%, and by the summer of 2022, it was 2.4% [50]. Among nursing-home residents in England, significantly lower hospitalization and mortality risks were also reported under the predominance of the omicron variant than under the previous variants [51], [52].

Whereas in 2021 – before omicron – there were still significant differences in hospitalization and case fatality rates between unvaccinated and vaccinated residents, with the predominance of omicron there was no longer any discernible effect of vaccination status. Unvaccinated residents no longer suffered more severe courses than vaccinated residents. Among staff, only sporadic cases of severe COVID-19 required hospitalization, and no deaths occurred from 2020 to 2022, with no discernible influence of vaccination status. 

It has been described previously that infections in elderly-care facilities are closely correlated with the incidence (infection rate) in the general population [53], [54], [55]. This was particularly evident in the omicron wave. The extensive protective and general hygiene measures, such as hand disinfection and nose-mouth protection for staff, distancing and isolation of infected residents, mandatory testing for visitors and staff, vaccination of residents, and mandatory vaccination of staff, could not prevent introduction of the virus into the facility and widespread dissemination, with a high infection rate among nursing-home residents and numerous outbreaks, but with a very low case fatality rate. 

As a consequence, not only the general population but also the risk group of nursing-home residents have built up a good immunity against SARS-CoV-2, even though immunocompetence decreases with age (immunosenescence). The severity of the disease and the case fatality rate are now low, even among nursing-home residents. In the facilities presented here, 97% of deaths in 2022 were due to causes other than COVID-19. 

Given the sometimes massive collateral damage of protective measures for nursing-home residents [6], [7], [8], [9], [10], [11], [12], [13], [14], [15], [16], a better balance between infection protection and quality of life for residents was already addressed in 2020 [56]. As early as the beginning of 2021, the question was raised as to which protective measures against SARS-CoV-2 continue to be necessary and appropriate in nursing homes for the elderly, and “considerations on the way back to normality” were made [57]. In view of the further development that has occurred by now – high vaccination and infection-rates and thus good immunity, low pathogenicity of the current virus variants also for nursing home residents – the considerations and proposals developed by this interprofessional and interdisciplinary working group no longer appear to be up-to-date and appropriate.

Meanwhile, SARS-CoV-2 – like other respiratory viruses – has become endemic. Some countries have therefore declared the end of the pandemic and lifted specific SARS-CoV-2 measures. Given the high immunity of the population and the low pathogenicity of the circulating virus – also among nursing-home residents – this should also be achieved in Germany [58]. Protective measures in nursing homes for the elderly that severely restrict people's right to self-determination and quality of life no longer seem justified, nor does the obligation to test visitors and staff members without reason, or the general FFP-2 mask requirement for staff members, without evidence for effectiveness [58], [23].

“Normality” must now also return to nursing homes; residents and visitors must regain their freedom of movement, daily activities, and, ultimately, their quality of life. Normality means: adequate protection of residents against infections in general (not exclusively SARS-CoV-2) by

- adherence to general hygiene rules such as coughing and sneezing etiquette, adequate ventilation of rooms [46], [47], [49], and keeping visitors and staff with symptoms of acute respiratory illness away (or at best under special protective measures such as distancing and masks),
- compliance with the recommendations of the KRINKO for the care of patients with infections, particularly the recommendation hygiene in homes [59], [60],
- adherence to STIKO vaccination recommendations not only against SARS-CoV-2, but especially against influenza and pneumococci [61].

The pandemic has highlighted the fact that the equipment and resources of long-term care facilities are limited. There is no professional or legal reason to continue the pandemic measures. The resources (staff, money) for these measures can be saved and would be better used elsewhere for the residents.

## Limitations

This is a small observational study in 5 nursing homes in one region. However, the low case fatality rate among nursing-home residents under the omicron variant is consistent with results from the general population in Germany and internationally, as well as in nursing homes in the UK [51], [52] and Munich, Germany [50]. All results presented are driven by the testing regimen; however, this did not change significantly between 2021 and 2022. The published hospitalization rate for the general population does not distinguish between “due to” or “with SARS-CoV-2”, which tends to overestimate this in the general population. However, the hospitalization rate for the SARS-CoV-2-positive nursing-home residents presented here may underestimate the severity of COVID-19 disease, because hospitalization is not considered in this at-risk group, if an appropriate living will is available. In contrast, the case fatality rate – defined here as death within 30 days of diagnosis of SARS-CoV-2 infection – is likely to be slightly overestimated [62].

## Notes

### Competing interests

The authors declare that they have no competing interests.

## Figures and Tables

**Table 1:**
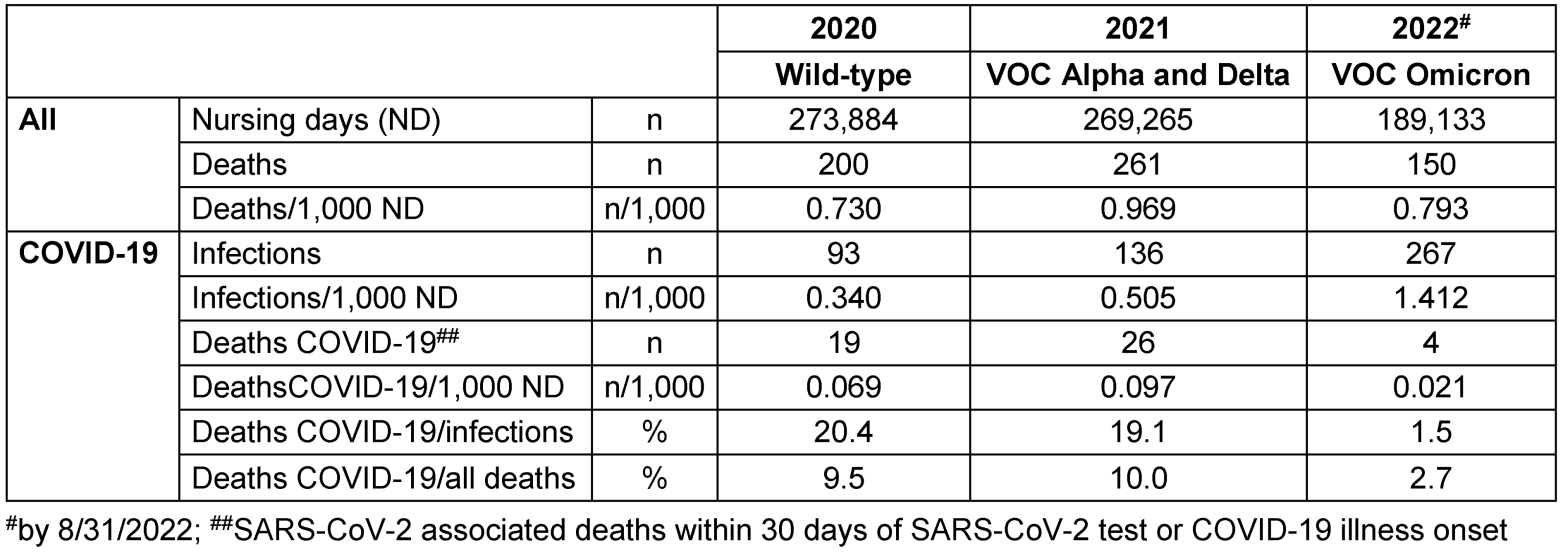
General structural data and mortality as well as SARS-CoV-2 infections and SARS-CoV-2 associated deaths among residents in 5 nursing homes in Frankfurt am Main, 2020 to August 31, 2022 – taking into account the predominantly circulating virus variant in each year

**Table 2:**
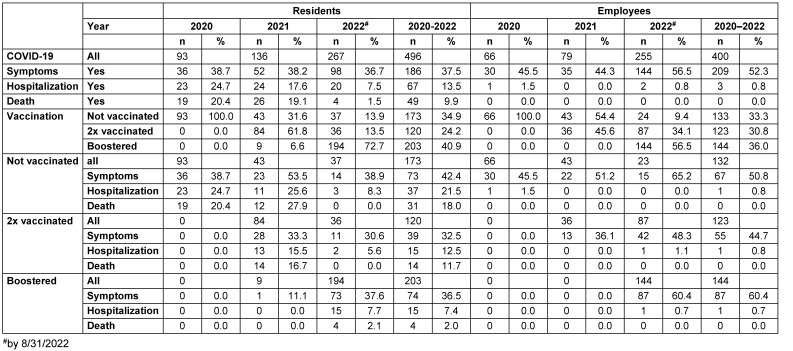
SARS-CoV-2-related morbidity and SARS-CoV-2-associated mortality of residents and staff overall and depending on the vaccination status in 5 nursing homes in Frankfurt am Main, 2020 to August 31, 2022
